# Prevalence and Causes of Elective Surgery Cancellations After Patients are Taken to the Operating Room: A Prospective, Cross-Sectional Study

**DOI:** 10.4274/TJAR.2024.231454

**Published:** 2024-02-28

**Authors:** Mustafa Soner Özcan, Eyyüp Sabri Özden, Filiz Alkaya Solmaz, Ayşe Kösem, Yiğit Akyol, Pakize Kırdemir

**Affiliations:** 1 Süleyman Demirel University Faculty of Medicine, Department of Anaesthesia and Reanimation, Isparta, Turkey

**Keywords:** Cancellation, elective surgeries, surgery scheduling, perioperative care, pre-operative assessment

## Abstract

**Objective:**

This study aimed to investigate the causes and prevalence of elective surgery cancellations in the operating room, and the clinical outcomes of affected patients.

**Methods:**

This prospective, cross-sectional study assessed the prevalence and causes of elective surgery cancellations once patients are in the operating room. A tertiary academic referral center hosted the study between January 2022 and January 2023. The study sample consisted of 7,482 adult patients scheduled for elective surgeries and taken to the operating room. The 7,415 completed procedures were in Group 2, whereas the 67 cancelled surgeries were in Group 1. Patients were divided into two groups on the basis of whether their surgeries were completed or cancelled. Factors such as age, American Society of Anesthesiologists (ASA) status, and surgical department were analyzed. The two groups were compared on the basis of age, ASA status, surgical department, and surgery time (month and day).

**Results:**

Elective surgery cancellations occurred in the operating room at a rate of 0.9%. Group 1 was substantially older than Group 2 (p<0.001). Group 1 had a larger number of ASA III patients (p<0.001). The department with the highest cancellation rate was ophthalmology (2.5%), followed by general surgery (2.1%), urology (1.5%), and ear, nose, and throat (1.4%). It was possible to avoid 59.7% of cancelations.

**Conclusion:**

The study revealed a 0.9% prevalence rate of elective surgery cancelations in the operating room. Older age and higher ASA status greatly influenced these cancellations. Optimized surgery scheduling and patient assessment processes may prevent many of these cancellation.

**Main Points**

• The study shows a 0.9% rate of elective surgery cancellations after operating room patient arrival.

• Elderly and high American Society of Anesthesiologists classes have a greater risk of elective surgery cancellations.

• Most cancellations in ophthalmology (2.5%), followed by gen surgery (2.1%) and urology (1.5%).

• 59.7% of cancellations are potentially avoidable, highlighting pre-op assessment flaws.

• Suggestions: better patient awareness, improved communication, and detailed preoperative checks.

## Introduction

Cancellations of scheduled elective surgeries reduce the operating room efficiency, increase the anxiety of the patients to be operated on as well as their families, lead to ineffective use of human resources and surgical supplies, and adversely impact overall health-related quality measures.^1,2^ Cancellations of scheduled elective surgeries also impose an extra financial burden on the healthcare system.^3^ The use of an organized multidisciplinary approach or a preoperative outpatient-based clinic has been associated with reduced rates of elective surgery cancellations.^1^

Various reasons, such as inadequate preoperative assessment, patient-related factors, underlying chronic diseases, and administrative or organizational issues, have been proposed for cancelling elective surgeries.^2,4^ Identifying the factors that cause the cancellation of elective surgeries is important in determining the appropriate strategies for improving patient satisfaction, operating room resources, and perception of quality of care.^5^ It has been stated that most of the reasons given for elective surgery cancellations could be prevented or potentially avoided via early assessment and meticulous planning and coordination before the patients to be operated on were hospitalized.^1,5,6,7^

The date an elective surgery is cancelled varies between the day the patient is notified of the scheduled surgery date and the day the surgery is scheduled to be performed. Unexpected cancellation of a surgery as late as the day the surgery is scheduled to be performed or even after the patient to be operated on is taken to the operating room has a more significant negative impact on hospital resources than cancellation of the surgery before the date it is scheduled to be performed.^6,8^ The emotional trauma experienced by patients and their families due to surgery cancellations is another negative result.^9,10^ Cancellation of elective surgery after the patient is taken to the operating room causes extra emotional distress in patients, in addition to unnecessary costs and ineffective use of hospital resources.^11,12^

There is limited data in the literature on the cancellation of elective surgeries once the patient is taken to the operating room. These cancellations can be reduced by determining the reasons for cancellation. In this context, this study was conducted to determine the prevalence and causes of cancellations of elective surgeries once the patient is in the operating room and the clinical course of the patients subject to surgery cancellation.

## Methods

### Study Design

The population of this prospective, cross-sectional study consisted of adult patients (aged 18 years or older) scheduled to undergo elective surgery between January 2022 and January 2023 at a tertiary academic referral center and taken to the operating room. The study protocol was approved by the Süleyman Demirel University Faculty of Medicine, Ethical Committee for Clinical Studies before the study (approval no: 3/33, date: 26.01.2022). The study was conducted in accordance with the principles outlined in the Declaration of Helsinki. Written informed consent was obtained from all patients.

### Study Setting

The tertiary academic referral center where the study was conducted is located in the Isparta Province in the Mediterranean Region of Turkey. According to 2022 statistics, the population of the province is 445,325. The center has 595 inpatient beds. A total of 34,658 medical and surgical patients were hospitalized at the center in 2022. The center has 18 operating rooms used by different departments. The regular working hours of the operating rooms are 8-AM to 4 PM on weekdays, except for public holidays.

### Patient Groups

Of the patients included in the study population, those who underwent emergency, obstetric, or minor elective surgeries that did not require preoperative filling out anaesthesiology assessment sheets and those who were scheduled outside the official working days, i.e., Saturday and Sunday, were excluded from the study. In addition, surgeries scheduled on the final operation list for that day or subsequently added to the operation list and cancelled before the patients were taken to the operating room were also not evaluated within the scope of this study. Of the remaining patients, those whose surgeries were cancelled by either the anaesthetist or surgeon after the patient was taken to the operating room and before or after the induction of general anaesthesia were included in Group 1, and those whose surgeries were completed were included in Group 2.

### Data Collection

All patients scheduled to undergo surgery were assessed preoperatively by the attending physician based on the type of surgery to be performed and the American Society of Anesthesiologists (ASA) class in the outpatient clinics of the Anaesthesiology and Reanimation Department. The surgeries were scheduled according to the results of the pre-operative assessment.

The follow-up data of the patients subject to surgery cancellation were obtained from the medical files of the patients available in the hospital or queried directly from the patients over the phone.

Patients’ demographic characteristics, i.e., age and gender; clinical characteristics, i.e., body mass index (BMI) and ASA class; and surgery details, i.e., the department where the surgery was performed/scheduled to be performed, and the month and the day the surgery was performed/scheduled to be performed were obtained from patients’ medical files and preoperative anaesthesiology assessment sheets and recorded. Comorbidities of patients subject to surgery cancellation reasons for cancellation and the clinical course of the patients subject to the cancellation during the 30 days after the cancellation were prospectively obtained and recorded into a structured worksheet designed for patients whose surgeries were cancelled.^11,12^

The reasons for cancellation were provided by the attending anaesthesiologists or surgeons and categorized as potentially avoidable or unavoidable based on their opinions. The guidelines of the European Society of Hypertension (ESH) and the European Society of Cardiology (ESC) were used to grade arterial hypertension.^13,14^ Patients with systolic blood pressures ≥180 mmHg or diastolic blood pressures ≥110 mmHg were considered to have stage 3 or higher hypertension, and their surgeries were cancelled. Potentially avoidable cancellations were those that could have been avoided if adequate preoperative assessment had been made or the hospital personnel had conducted the necessary communications before the scheduled date and time of the surgery. The definitions of potentially avoidable or unavoidable surgery cancellations were included in the structured worksheet for the attending anaesthesiologists or surgeons who are to fill out the worksheet.

### Statistical Analysis

The prevalence of elective surgery cancellations after the patient was taken to the operating room was determined as the primary outcome of the study. The study’s secondary outcomes included the factors potentially contributing to the elective surgery cancellations such as the department where the surgery was performed/scheduled to be performed and the month and day the surgery was performed/scheduled to be performed. To ensure the integrity and validity of the study findings, statistical analyses were performed using Jamovi project 2.3.28 (Jamovi, version 2.3.28, 2023, retrieved from https://www.jamovi.org) and JASP 0.17.3.0 (Jeffreys’ Amazing Statistics Program, version 0.17.3.0, 2023, retrieved from https://jasp-stats.org) software packages, which are recognized for their robust statistical capabilities.

Before conducting any inferential statistical tests, the normal distribution characteristics of continuous variables were rigorously analyzed using the Shapiro-Wilk, Kolmogorov-Smirnov, and Anderson-Darling tests. This step was pivotal in determining the statistical tests for subsequent analyses. The Mann-Whitney U test, known for its non-parametric nature and efficacy in comparing medians, was used to compare variables not conforming to a normal distribution.

Categorical variables, such as the department where the surgery was performed/scheduled to be performed and the month and day the surgery was performed/scheduled to be performed, were analyzed using Pearson’s chi-square test, a test used as a standard in medical research for comparing proportions among independent groups. The Fisher-Freeman-Halton test was used in tables with more cells than in a 2x2 table. Probability (*P*) statistics of ≤0.05 were deemed to indicate statistical significance, in line with conventional standards in medical research to minimize Type I errors.

## Results

The study sample consisted of 7,482 patients. Of these patients, 67 whose surgeries were cancelled were included in Group 1. Accordingly, the prevalence of cancellations of elective surgeries once the patient was in the operating room was 0.9%. The remaining 7,415 patients were included in Group 2.

The distribution of patients’ demographic and clinical characteristics by patient groups is shown in Table 1. Group 1 was significantly older than Group 2 (*P* < 0.001). There was no significant difference between the groups in median BMI values (*P*=0.523). On the other hand, the number of patients categorized as ASA III was significantly higher in Group 1 than in Group 2 (*P* < 0.001). In parallel, the number of patients with the ASA 1 class was significantly higher in Group 2 than in Group 1 (Table 1).

**Figure 1:**
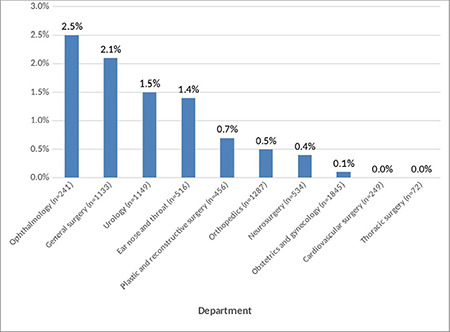
Distribution of the cancellation rates based on the surgical departments.

There were also significant differences between the groups in terms of the department where the surgery was performed/scheduled to be performed, and the month and day the surgery was performed/scheduled to be performed (*P* < 0.05) (Table 2). The department with the highest cancellation rate of elective surgeries was ophthalmology (2.5%), followed by general surgery (2.1%), urology (1.5%), and ear, nose, and throat (1.4%) (Figure 1).

**Figure 2:**
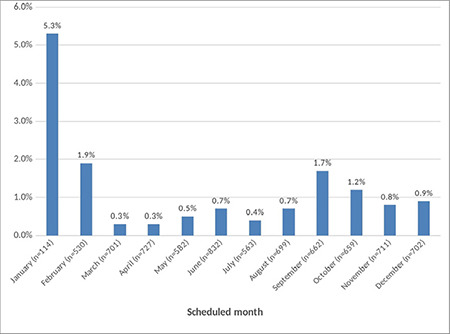
Distribution of the cancellation rates based on the scheduled months.

**Figure 3:**
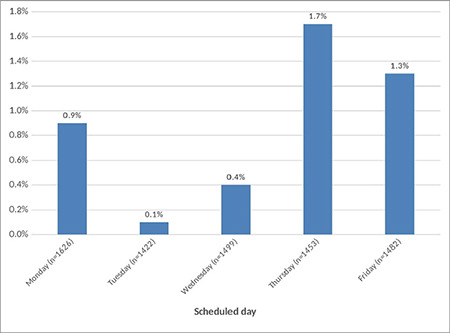
Distribution of the cancellation rates based on the scheduled days.

The month with the highest cancellation rate of elective surgeries was January (5.3%), followed by February (1.9%) and September (1.7%) (Figure 2). In terms of the days when elective surgeries were cancelled, the cancellation rates of elective surgeries scheduled for Tuesday and Wednesday were lower than those scheduled for other days of the week (Figure 3).

The reasons for elective surgery cancellations are given in Table 3. Stage 3 or 4 hypertension (46.3%) and smoking on the day of the scheduled surgery (26.9%) were the most frequently cited reasons for elective surgery cancellations. Of the causes given for the cancellations of elective surgeries, 59.7% were evaluated as potentially avoidable.

Regarding the clinical course of the patients subject to the cancellation during the 30 days after the cancelation 53 (79.0%) patients underwent surgery after the cancellation of the surgery. Most (98.1%) of these surgeries were performed in the same hospital.

## Discussion

The study findings show that less than one percent of the elective surgeries were cancelled once the patients were taken to the operating room. The elective surgery cancellation rates were found to be higher in the ophthalmology, general surgery, urology, and ear, nose, and throat departments than in the other departments. However, a preliminary analysis of the factors impacting elective surgery cancellations indicated the necessity for a detailed analysis. More than half of the reasons given for elective surgery cancellations were categorized as potentially avoidable factors. Most of these factors were associated with the patients’ medical conditions. In this context, increasing the awareness of patients for surgery, improving the communication between patient and physician as well as between patient and hospital administration, and preoperatively assessing patients for elective surgeries are likely to reduce elective surgery cancellation rates.

There are only a few studies in the literature on elective surgery cancellations after patients are taken to the operating room. In two of these studies with seven- and eight-year study periods, surgery cancellation rates after the patients were taken to the operating room were reported as <0.01% and 0.21%, respectively.^11,12^ In comparison, in this study, which had a one-year study period, the prevalence of cancellations of elective surgeries once the patient was in the operating room was 0.9%. There were also significant methodological differences between this study and the other two studies. Hori et al.^11^ evaluated 30 patients whose surgeries were cancelled after they were taken to the operating room and prepared for general anaesthesia. Eighteen of these patients had their surgeries cancelled before the induction of general anaesthesia and 12 after the induction of general anaesthesia. The primary reasons given for the cancellations after the induction of general anaesthesia were anaphylactic shock (n = 3) and arrhythmias (n = 4). In the study by Perroca et al.^15^, surgery cancellations were stratified according to whether they occurred before the preparation and arrangement of the operating room or after the operating room was prepared and anaesthetic-surgical procedures were started. In comparison, in this study, surgery cancellations were not stratified as such.

Hori et al.^11^ and Chang et al.^12^ reported surgery cancellation rates after the induction of general anaesthesia as 40% and 12.7%, respectively. In comparison, in this study, none of the 67 surgery cancellations were made after the induction of general anaesthesia. Surgery cancellations after initiating general anaesthesia are rare and are primarily due to unexpected and unpredicted changes in the patient’s clinical condition.^11,12,15^

Fitzsimons et al.^16^ investigated 43 cardiac surgery cancellations made after the patients were taken to the operating room and before the initiation of surgical incisions. These 43 surgery cancellations constituted 0.84% of all surgeries conducted during the four-year study period. Similar surgery cancellation rates have been reported in other studies.^17,18^ The findings of these studies have not been compared with those of the studies that specifically addressed cardiac surgery cancellations due to technical differences specific to heart surgeries.

Stratifying the reasons for surgery cancellations as potentially avoidable or unavoidable may not be a precise method.^3,7,12^ In fact, Hori et al.^11^ categorized 36.7% of the reasons given for surgery cancellations before the induction of general anaesthesia as potentially avoidable, stating that they could have been prevented by improving the pre-operative assessment of the patients in terms of their medical problems, 59.7% of the reasons given for surgery cancellations before the induction of general anaesthesia in this study were categorized as potentially avoidable. In different studies, the rate of potentially avoidable reasons among the reasons given for elective surgery cancellations before the induction of general anaesthesia has been estimated to be between 60% and 70%.^3,7,15^ It is not an easy task to compare these studies because of the heterogeneities in patient groups and methodologies. However, detailed preoperative assessment of patients is the most effective measure to prevent surgery cancellations.^11^

Previous studies grouped the causes of surgery cancellations as patient-related, facility-related, and surgery-related.^2,3,4,5,7,19,20^ The characteristics of patients, hospital facilities, medical and administrative staff, and the socioeconomic status of patient populations have been cited as the primary components of patient-related, facility-related, and surgery-related causes resulting in surgery cancellations.^21^ We did not use such stratifications due to the narrower focus of the study, which addressed only the cancellations made once the patient was taken to the operating room. Becker et al.^22^ investigated the non-medical risk factors that lead to postponing elective surgeries. They found that advanced age, retirement, and nursing home residence were risk factors for surgery cancellation and rescheduling. Along these lines, in this study, the patients whose surgeries were cancelled were significantly older, and a higher number of patients whose surgeries were cancelled were categorized as ASA III compared with the patients whose surgeries were completed as scheduled. Although the patients whose surgeries were cancelled were significantly older than those whose surgeries were completed as scheduled, the fact that their median age was below 65 may be a confounding factor. We did not separately investigate the impact of each demographic and clinical characteristic on cancellation rates. Nevertheless, we found that the reasons for surgery cancellations were mainly related to the patients’ medical conditions and may thus be considered patient-related reasons.

According to the current study, smoking and uncontrolled hypertension were the most frequent causes of cancellations for all elective surgeries. The management of hypertension during the perioperative period has become increasingly crucial considering the growing number of surgeries performed worldwide and the large number of hypertensive patients.^23^ In addition, preoperative hypertension raises perioperative risks, which may cause anaesthesiologists to hesitate during the induction of anaesthesia.^24^ The decision to proceed with surgery in patients with uncontrolled hypertension before induction of anaesthesia has been a medical dilemma for many years.^25,26^ Guidelines from the ESC and the ESH state that if blood pressure is 180 mmHg systolic and 110 mmHg diastolic, elective surgery should not be cancelled.^23^ In a prospective comparative study, Soni et al.^27^ found that inappropriate cancellations due to hypertension decreased significantly over the years (from 1.37% to 0.05%) in keeping with the recommendations in the guidelines. In accordance with recently released guidelines, all cancellations in this study involving uncontrolled hypertension occurred in patients with stages 3 and 4. A comparable number of patients were assessed in this study and the one by Soni et al.^27^. On the other hand, cancellation rates due to uncontrolled hypertension may have been higher in our center because cancellation times were different between the two studies. This difference can be explained by patients’ non-compliance with treatment regimens, disruption of periodic check-ups, and sociocultural context.

Preoperative smoking is associated with impaired wound healing and many serious complications involving the pulmonary, cardiovascular, and neurological systems.^28,29^ It is most likely the result of cumulative chronic and acute toxic effects from inhalation. Consequently, if a patient smokes before an elective procedure, anaesthesiologists may cancel the procedure. In this study, the reason for most of the cancelled cases was smoking. Based on evidence from randomized controlled trials, guidelines recommend smoking cessation for at least 4 weeks before elective surgeries.^30^ This may not be possible in the daily routine. Although preoperative smoking is considered a preventable cause with education about its potential harms in the perioperative period, smoking may not be prevented because it is related to the patient’s sociocultural and addiction status. Patients may smoke covertly prior to surgery, and some may even admit smoking before the induction of anaesthesia.

The study’s prospective design was its primary strength because it allowed a more accurate data collection process. This study did not address cancellations made before the patients were taken to the operating room. Thus, the study’s single-center design, along with the fact that only the cancellations made after the patients were taken to the operating room were taken into consideration, may limit the generalizability of this study’s findings to other settings. In addition, the significance level of the statistical analyses conducted according to the department where the surgery was performed/scheduled to be performed, and the month and the day the surgery was performed/scheduled to be performed may be questioned due to the smaller sizes of each subgroup. Lastly, we had difficulty explaining the unexpectedly higher surgery cancellation rates observed in January, which is likely associated with administrative issues.

## Conclusion

The prevalence of elective surgery cancellations after the patients were taken to the operating room was less than one percent (0.9%). Most surgery cancellations were related to patients’ medical conditions and lack of surgical awareness. More than half of the reasons given for surgery cancellations were categorized as potentially avoidable. Therefore, improving the surgical awareness of patients, establishing effective communication with patients, and preoperatively assessing patients for elective surgical procedures are likely to reduce elective surgery cancellation rates after the patients are taken to the operating room.

## Figures and Tables

**Table 1:**
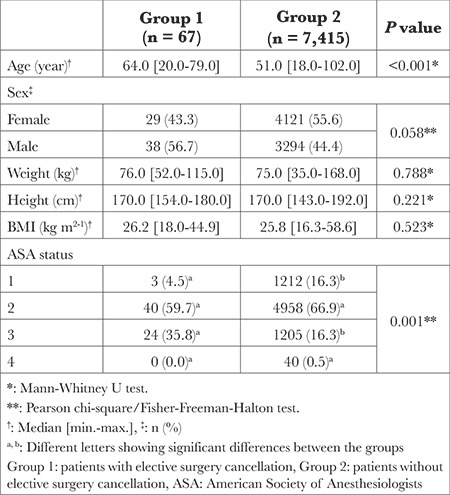
Demographic and Clinical Characteristics of the Patients with and Without Elective Surgery Cancellation

**Table 2:**
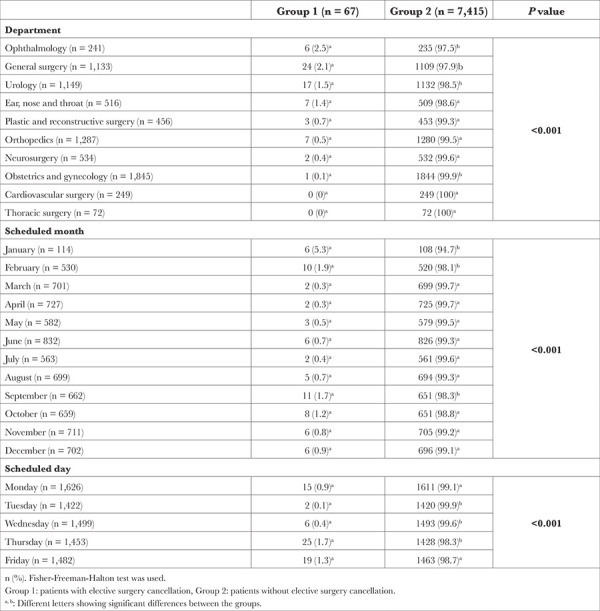
Cancellation Rates of the Groups Based on the Surgical Department, the Scheduled Month and Day

**Table 3:**
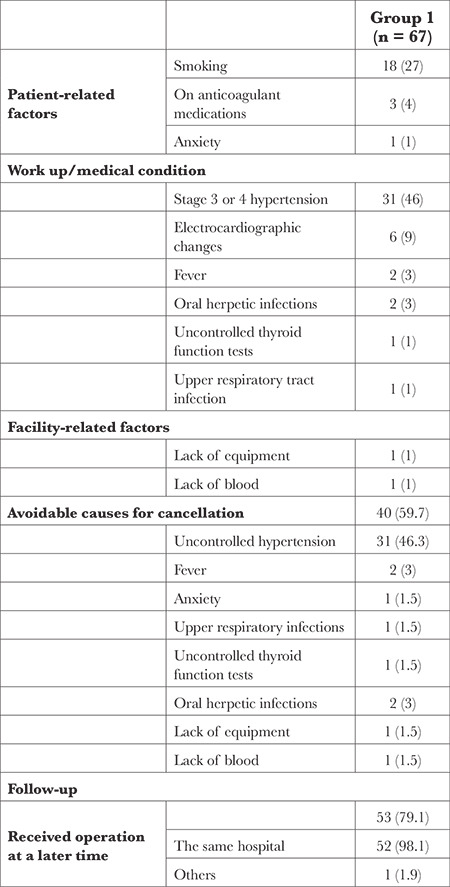
Reasons for Surgery Cancellation
